# Properties of Fixed-Fixed Models and Alternatives in Presence-Absence Data Analysis

**DOI:** 10.1371/journal.pone.0165456

**Published:** 2016-11-03

**Authors:** Aleksi Kallio

**Affiliations:** 1 Department of Computer Science, Aalto University, Espoo, Finland; 2 CSC - IT Center for Science, Espoo, Finland; Queen’s University Belfast, UNITED KINGDOM

## Abstract

Assessing the significance of patterns in presence-absence data is an important question in ecological data analysis, e.g., when studying nestedness. Significance testing can be performed with the commonly used *fixed-fixed* models, which preserve the row and column sums while permuting the data. The manuscript considers the properties of fixed-fixed models and points out how their strict constraints can lead to limited randomizability. The manuscript considers the question of relaxing row and column sun constraints of the fixed-fixed models. The Rasch models are presented as an alternative with relaxed constraints and sound statistical properties. Models are compared on presence-absence data and surprisingly the fixed-fixed models are observed to produce unreasonably optimistic measures of statistical significance, giving interesting insight into practical effects of limited randomizability.

## Introduction

Binary presence-absence datasets encode information on species occurrence over different sites. The datasets are essentially simple matrices of ones and zeros, but they form the basis for analyses of more complex phenomena, such as correlation between occurrences of taxa [1], nestedness [2, 3] or niches [4]. An essential part of quantitative analysis is assessing the statistical significance of the results. In the case of presence-absence data, the task of significance testing is often difficult due to the high-level structure of the data matrix, described by the row and column sums. Those sums indicate the number of ones in corresponding rows and columns, and are collectively referred to as *margin sums*. The distribution of margin sums can often be skewed, which causes problems for typical statistical tests.

Currently ecological literature often suggests that margin sums should not explain interesting patterns, but instead be included in the null model [5–8]. Preserving margin sums retains differences of species richness over sites and occurrence frequencies among species [9]. The way in which null models should be constructed to preserve margin sums has been in the center of the debate since the null model approach was introduced by [10]. For a good overview of the debate, the reader is referred to [5, 8, 11]. The challenge of margin sum aware significance testing has lead to a wide collection of suggested null models [2, 3, 9, 12–16].

## Analysis

Null model tests for presence-absence data follow the idea of classical statistical randomization tests. Adapting from [9], the practical steps could be summarized as:

1. Define a measure that with a single number (statistic) describes the strength of pattern in matrix (e.g., co-occurrences)
2. Measure the actual (observed) data matrix
3. Randomize the data matrix using the null model and measure it again
4. Repeat step 3 many times to generate a set of statistics from randomized data (null distribution)
5. Use statistical inference to interpret the original statistic against the set of statistics from randomized data

If the original statistic is similar to the randomized counterparts, then the result is not considered significant.

### Fixed-fixed models and checkerboard units

For significance testing that takes row and column sums into account, an obvious null model is to preserve the sums while randomly permuting the ones inside the matrix. This so-called *fixed-fixed model* (fixed row sums, fixed column sums, FF) is widely adopted in presence-absence studies. Random matrices that satisfy fixed-fixed constraints can be produced with a *sequential swap procedure*. The procedure starts with the original matrix and performs swaps of cell values to produce a new random matrix.

**Fig 1:**
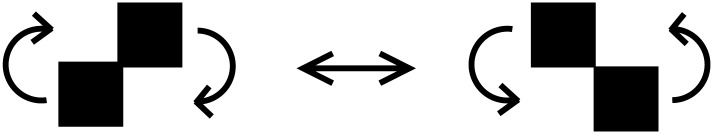
Checkerboard units of 2 × 2 values with ones shown in black and zeros shown in white. The schematic description shows how by swapping elements inside the unit it is possible to switch between the two units without changing margin sums.

Swaps operate on row and column pairs with a specific organization of values, shown in Fig 1. These row and column pairs are called checkerboard units or switch boxes [17, 18]. Given matrix *M*, checkerboard units are formalized by Definition 1.

**Definition 1.** *Denote values on a pair of rows and a pair of columns with a* = *M*(*i*_1_, *j*_1_), *b* = *M*(*i*_1_, *j*_2_), *c* = *M*(*i*_2_, *j*_1_) *and d* = *M*(*i*_2_, *j*_2_). *A pair of rows* (*i*_1_, *i*_2_) *and a pair of columns* (*j*_1_, *j*_2_) *contain a checkerboard unit if and only if* *a* = *d*, *b* = *c* *and* *a ≠ b*.

Ryser, among others, has shown that all matrices with identical margin sums can be produced with swap operations [19]. When the sequential swap procedure is implemented correctly, the consecutive matrices form a Markov Chain that has uniform stationary distribution [11, 20]. To summarize, the sequential swap procedure is a good way of sampling matrices at random from the uniform distribution of all matrices that have identical margin sums.

It has been argued by several authors that limiting the randomization process to strictly same margin sums might not give realistic results [5, 21–23]. If only few perturbations are possible, then the significance test will compare against randomized matrices which are very similar to the original one and results of the test will be extremely conservative.

The concern of insufficient perturbation is especially valid when the sequential swap procedure is applied to nested data. Definitions of nestedness vary in details, but all share the basic idea of all columns (and rows) having a superset-subset relationship [3, 24].

Every checkerboard unit is a breach of the subset-superset pattern: if a checkerboard unit exists for rows (*i*_1_, *i*_2_) of matrix *M*, there cannot be subset-superset relationship between $M_{\left(i_{1},\cdot \right)}$ and $M_{\left(i_{2},\cdot \right)}$, and this holds for columns also. If all rows (and columns) are perfectly nested in the sense of superset-subset relationships, then the matrix cannot contain any checkerboard units and it is not possible to permute it with swap operations.

### Properties of fixed-fixed models

The fixed-fixed models are implemented with the sequential swap procedure, which produces random matrices with strictly same margin sums. Given row sums *R* and column sums *C*, the sequential swap procedure samples in random from the set of matrices $M^{R,C}$. However, not much is known about the set matrices $M^{R,C}$, such as the number of matrices in the set. As discussed, there are concerns that matrices in $M^{R,C}$ resemble the original matrix too much. Because the space of matrices $M^{R,C}$ is not well understood, it is possible that there are also other undesirable aspects related to it.

To understand the implications of fixed-fixed margin sum constraints, the following example data matrix is considered.

$$M=\left(\begin{array}{ccc} 1 & 0 & 1\\ 0 & 1 & 0 \end{array}\right)$$

Matrix *M* has margin sums *R* = < 2, 1 >, *C* = < 1, 1, 1 >. It corresponds to a case where there are two species, the first of them being present most of the time and the second being absent most of the time. The three sites have one of the two species present. When asked to produce a random matrix that follows this basic pattern, one could argue that a probable outcome could be

$$M^{\prime }=\left(\begin{array}{ccc} 1 & 1 & 1\\ 0 & 0 & 0 \end{array}\right)$$

This matrix differs from *M* by two cells: the first species is present also on the second site and the second species is also absent on the second site. These two cells and especially the absence on second row could be considered as chance occurrences in the original matrix *M* [25].

In the fixed-fixed margin sum model, matrix *M*′ has probability zero, because margin sums of *M* and *M*′ differ. The three matrices that have strictly same margin sums all have both of the unlikely values, but in a different column. Preserving such a quirk of the original dataset would not necessarily be desirable. Row and column sum constraints are all linked together, creating situations that could be described as “lock-ins”. It is useful to consider alternative models that relax strict margin sum constraints and see how “lock-in free” models perform.

### Rasch models as alternative

The advantage of fixed-fixed model is its simplicity. If one needs to account for margin sums, then it is easy to justify a model that does not change them at all. The central question in designing a relaxed alternative model is: in what way constraints should be relaxed to not introduce any hidden biases? Here a useful tool is the information theoretic concept of *maximum entropy principle*. The principle states that the null model should maximize entropy within the given constraints [26]. Maximum entropy distribution could be described informally as the most random distribution. It is noteworthy that the uniform distribution produced by the sequential swap procedure is the maximum entropy distribution under fixed-fixed constraints, or in other words, the procedure is the statistically correct implementation for fixed-fixed constraints.

The motivation is to find a model that replaces strict constraints with relaxed stochastic constraints that maintain the margin sums well enough. It is important not to use too relaxed constraints for the null model, as it leads to abundant false positives [9]. There are various models for stochastically margin sum constrained random matrices, most importantly the proportional-proportional models [6, 22]. They are not derived under the maximum entropy principle, making them ill-suited for current discussion of properties and for comparison with the fixed-fixed models.

An alternative family of models with stochastic constraints for margin sums are the Rasch models, which were first widely used in psychometrics. To the author’s knowledge they have not been applied to ecological presence-absence data before. Rasch models preserve statistical expectations instead of exact values: they constrain expected margin sums in the null distribution to equal the margin sums in the original matrix. De Bie, among others, has demonstrated how expected margin sums can be formalized to obtain a convenient computational sampling method [26]. In a Rasch model, each cell of the matrix is initialized to value 0 and set to value 1 with probability defined by Eq (1).

$$p_{i,j}=\frac{exp(\lambda_{j}+\mu_{i})}{1+exp(\lambda_{j}+\mu_{i})}$$ (1)

In Eq (1), *p*_*i*, *j*_ is the probability for cell in row *i* and column *j* to get value 1. The parameters λ_*j*_ and *μ*_*i*_ are column and row specific parameters that are fitted to the original data matrix. For details on parameter fitting, see [26]. Is is easy to see that given λ and *μ*, cell specific probabilities are independent. Generating matrices is straightforward, as parameters need to be fitted only once and then cell values can be generated independently. Eq (1) is derived from the row and column sum constraints by using the maximum entropy principle. See, e.g., [26] for description of the steps needed to turn the constraint equations into Eq (1).

Looking back at example matrix *M*′, which was not achievable by sequential swap procedure:

$$M^{\prime }=\left(\begin{array}{ccc} 1 & 1 & 1\\ 0 & 0 & 0 \end{array}\right)$$

Under the Rasch model, the most likely matrix for margin sums *R* = < 2, 1 >, *C* = < 1, 1, 1 > is *M*′. All of the matrices that the sequential swap procedure produces have high probability under the Rasch model, as well.

### Properties of fixed-fixed and stochastic constraints

Fixed-fixed constraints can in some cases rule out matrices that are not so distinct from the other matrices that adhere to the constraints. The important follow-up question is: does this happen with large datasets and if it does, does it matter for significance testing? To demonstrate “lock-in” effect in larger matrices, the following experiment is planned. In the experiment a sequence of swap operations is performed on a presence-absence matrix called NOW MN5, which was extracted from the NOW database of fossil mammals [27]. Please refer to Section Datasets and methods for details of the NOW MN5 matrix.

Correlation analysis is used to demonstrate how the sequence of swap operations breaks the structures within the matrices. The progress of the sequential swap procedure is tracked by performing swaps and counting strong positive pairwise correlations of sites with the procedure recommended by [1]. Correlation counts are recorded after every 10 attempted swaps. The number of correlations is used as a measure for structure, so that low count of correlations corresponds to lack of structure. The swap procedure is run for total of 2000 attempted swaps and total of 10 swap chains are produced.

**Fig 2:**
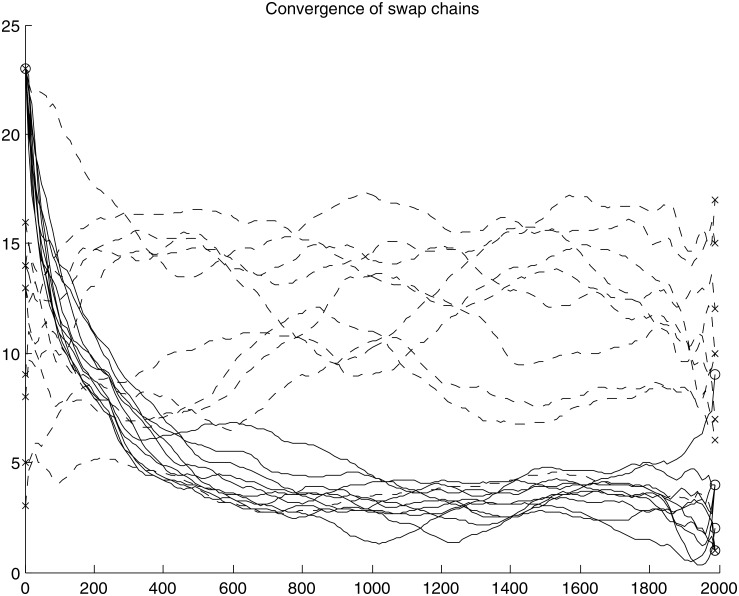
Count of correlations during sequential swaps. Each line shows the fluctuation in correlation count during a single chain of swaps. Solid lines show swap chains that start from the original dataset on the left and terminate in result datasets on the right (marked with “o”). Dashed lines show chain of swaps for 10 Rasch randomized datasets (marked with “x”). Correlation counts were recorded every 10 attempted swaps. Lines are smoothed for readability with moving window of 50 counts.

For comparison, a Rasch model is initialized with the NOW MN5 matrix and it is used to generate 10 random matrices that have same expected margin sums as the NOW MN5 matrix. One swap chain is run and recorded for each of those matrices, producing a total of 10 swap chains. All of the 20 chains are shown in Fig 2.

From Fig 2 it can be seen that the two sets of chains are strikingly different. Chains that were started from the Rasch randomized matrices seem to have correlation counts that randomly wander in close neighborhood of the original value. Swap operations obviously do not have any major effect on the matrices generated by the Rasch model. The result is different when swap operations are applied to the original NOW MN5 matrix. The sequential swap procedure preserves strict margin sums and one could assume that it would retain more structure than the Rasch model. This was not the case, but instead swaps quickly removed structure from the matrix and converged to a level of less than 5 correlations. For comparison, the initial Rasch matrices that were produced from the same NOW MN5 matrix mostly measured from 10 to 15 correlations. The unexpected observation is that despite more conservative constraints, the sequential swap procedure was in practice less conservative. It is also noteworthy that the sequential swap procedure converged to a different level when it was started from the original NOW MN5 matrix, compared to being started from Rasch randomized matrices, which have similar margin distribution. This observation will be examined further in the next experiment.

The visual observations from Fig 2 can be validated using the non-parametric Kolmogorov-Smirnov two sample test to compare distributions. The three distributions compared are: swapped distribution (“o” symbols on the right), Rasch distribution (“x” symbols on the left) and swapped Rasch distribution (“x” symbols on the right). The null hypothesis is that samples are drawn from two different distributions and the null hypothesis is rejected at significance level *p*′ < 0.05. To account for multiple comparisons, Bonferroni correction is applied to obtain significance threshold of *p* < 0.05/3 = 0.017. *p*-values are given in Table 1.

**Table 1:** *p*-values for Kolmogorov-Smirnov tests between the pairs of distributions. Significance threshold is 0.017.

Rasch and swapped Rasch	*p* = 0.313
swapped and swapped Rasch	*p* = 0.001
swapped and Rasch	*p* = 0.001

Kolmogorov-Smirnov test between Rasch and swapped Rasch distributions is the only one where null hypothesis is not rejected and the assumption of samples coming from the same distribution is maintained. When the number of correlations is used as a measure of structure, swap operations on Rasch generated matrices do not produce any significant changes to structure. This conclusion can also be arrived theoretically, as swapping does not change the probability of matrix under the Rasch model.

Swapped and swapped Rasch distributions were found to be different, supporting the visual observation that swaps converged differently, depending on the initial matrices. Finally, swapped and Rasch distributions were also found different, suggesting that the two methods produce significantly different results when applied to the original NOW MN5 dataset.

The matrices produced by the sequential swap procedure are a subset of the matrices produced by a Rasch model. This fact however tells little about the actual content of the matrices. The bulk of the probability mass under Rasch model can be located in matrices that are not produced by the fixed-fixed model. The correspondence between constrains of a model and the null distribution it produces can run against intuition. Generalizability of this observation is studied next, as well as the potential causes for the unexpected behavior.

### Datasets and methods

It was shown that fixed-fixed models can be less conservative than Rasch models. To check if this observation can be generalized to a larger number of datasets and statistics, the models are next used for significance testing on presence-absence datasets.

Three different datasets are used in the experiment: NOW, NOW MN5 and Vanuatu. Datasets NOW and NOW MN5 are imported from the NOW database of fossil mammals [27]. The database was chosen as previous experiments with it sparked the concerns about undesired performance. Besides the whole NOW dataset, sites from MN zone 5 only are selected to produce NOW MN5 dataset without the strong global time dependent structure of the whole NOW database [1]. Furthermore, NOW datasets are harmonized by including only large land mammals and excluding species with less than 10 occurrences.

Dataset Vanuatu is the classic Vanuatu (formerly New Hebrides) avifauna dataset that is used for comparison. The data matrix is available in a table form from the original publication of [28]. The data table was written down from the journal article and used without any preprocessing. The same dataset was used by [10] when they presented the null model approach for the first time. They argued against the community rules proposed in [29] and discussed how similar patterns can be achieved by arranging presences by random and following simple rules which correspond to fixed-fixed constraints of this manuscript.

The null models are used to derive significance measures for a selection of different statistics. The sequential swap implementation is based on the procedure of [17]. During the computation swaps were attempted 1000 times the number of ones, which is 1000 times more than [17] recommended as minimum for convergence. The Rasch model implementation is based on the procedure of [26].

The significance measures are computed for both local and global statistics. Local statistics measure the strength of local patterns that govern only part of the dataset, where as global statistics measure the strength of patterns that govern the whole dataset.

Cluster structure and checkerboard unit count are used as global statistics. Clustering of the dataset was done with K-means method. The number of species clusters *k* was set to 2, 5 and 10. As a measure of error the sum of distances to cluster centroid is used, separately for all values of parameter *k*. The method is robust in a sense that the cluster structure or the corresponding measure of error is not dependent of row and column order.

Checkerboard unit count is a concept related to nestedness. Different definitions for nestedness have been proposed and currently there is no agreement on the gold standard [3]. Here checkerboard unit count is used as a proxy statistic for nestedness. It has the advantage of being robust (not dependent on row and column order) and computationally well defined. For these reasons, checkerboard units are well suited for repeated randomization tests.

To measure local structure in the datasets, intra-species relationships were analyzed. For this purpose, the count of pairwise species correlations are measured, including positive, negative and two-tailed correlations, following [1]. Correlation is decided using Fisher Exact Test. Significant results are given for *p* < 10^−6^ and were not adjusted for multiple hypothesis testing to keep them comparable. The number of positive, negative and two-tailed correlations are reported.

Matlab source code for the experiments is distributed under the MIT license and available from https://github.com/akallio1/marginsums.

### Significance testing results

Fixed-fixed model and Rasch model were used for significance testing on presence-absence datasets. In the experiment, a set of statistics is calculated from presence-absence binary matrices and compared to values derived from randomized datasets that share the same marginal sums as the original datasets (n = 1000, repeated for both null models).

Results of the experiment are given in Table 2. When interpreting the results, correlations counts are measures of structure, i.e., larger value means stronger structure, and all other statistics are measures of error, so that smaller values mean stronger structure. Sum of negative and positive correlation counts is not necessarily equal to the count of two-tailed (negative and positive) correlations due to the formulation of p-value cutoff: for details we refer to [1]. *p*-values are defined as the empirical probability of observing structure at least as strong from the randomized data.

**Table 2:** Statistics for original datasets and randomized datasets with both the fixed-fixed null model and Rasch null model. Median statistic over all randomizations is reported for randomized data. *p*-values are defined as the empirical probability of observing statistic at least as extreme from the randomized data. For each dataset dimensions are given together with the fill ratio, i.e., the ratio of matrix cells with value 1.

Dataset NOW MN5 (254 rows and 24 columns with fill ratio of 0.076)					
	Original	fixed-fixed model		Rasch model	
	Statistic	Statistic	p	Statistic	p
Checkerboard unit count	54728	53634.5	1	47403	0.982
Correlation count (pos)	23	3	0.001	9	0.013
Correlation count (neg)	0	0	1	0	1
Correlation count (both)	23	3	0.001	9	0.013
Clustering error (k = 2)	331.53	342.61	0.001	329.58	0.562
Clustering error (k = 5)	262.64	292.54	0.001	276	0.108
Clustering error (k = 10)	202.45	242	0.001	225.86	0.01
Dataset NOW (3207 rows and 223 columns with fill ratio of 0.016)					
	Original	fixed-fixed model		Rasch model	
	Statistic	Statistic	p	Statistic	p
Checkerboard unit count	55385342	53819460.5	1	52790464.5	0.998
Correlation count (pos)	2355	233	0.001	370	0.001
Correlation count (neg)	7	0	0.001	0	0.001
Correlation count (both)	2362	232	0.001	370	0.001
Clustering error (k = 2)	10269.63	10521.75	0.001	10459	0.018
Clustering error (k = 5)	9085.61	10040.86	0.001	9998.54	0.001
Clustering error (k = 10)	8209.21	9669.95	0.001	9637.17	0.001
Dataset Vanuatu (56 rows and 28 columns with fill ratio of 0.564)					
	Original	fixed-fixed model		Rasch model	
	Statistic	Statistic	p	Statistic	p
Checkerboard unit count	14702	14065	1	11685.5	0.99
Correlation count (pos)	25	7	0.001	12	0.042
Correlation count (neg)	0	0	1	0	1
Correlation count (both)	21	5	0.001	10	0.054
Clustering error (k = 2)	215.37	216.49	0.108	208.96	0.831
Clustering error (k = 5)	154.88	163.92	0.001	153.7	0.581
Clustering error (k = 10)	110.33	125.94	0.001	116.16	0.159

The major trend in *p*-values is that the two models give very different results, with swaps being less conservative. Here the same effect is observed as in the previous experiment: when using statistics as indicators of structure, the fixed-fixed model generates random matrices that share surprisingly little with the original matrix. This observation has two immediate implications. First, the choice of null model matters, as in most cases the *p*-value given by a fixed-fixed model is below the typical threshold of 0.05, where as the *p*-value given by a Rasch model is above. Second, the results would favor the skeptical side on the long running argument initiated by [10]. Connor and Simberloff used the fixed-fixed type of model in their argumentation to show how random arrangement of values can lead to patterns that follow the assembly rules [10]. The results in Table 2 indicate that Rasch models could provide even stronger support for the case, while fulfilling the requirement of random arrangement by having independent cell probabilities, given the margin sums.

Looking at Table 2, margin sums explain most of the global structure, nestedness and cluster structure. Clustering errors in original data and randomized data under either model are relatively close. However the difference is systematically such that original data has stronger structure. Hence structures are considered significant in most cases with fixed-fixed model and in some cases with Rasch model. The null models show that data contains a statistically significant global signal, but it is weak and possibly irrelevant. There is also a difference between the models: the Rasch model generates data that resembles original data more closely. For this reason the Rasch model is able to discern between different values of *k*. Results from the Rasch model suggest that 2 or 5 clusters cannot capture any significant structure in the NOW MN5 dataset. NOW dataset has stronger cluster structure, clearly related to different MN zones inside the dataset. Vanuatu dataset seems to have no strong cluster structure.

Checkerboard unit counts in Table 2 indicate that all datasets are significantly anti-nested. In all cases the randomized datasets have less checkerboard units, under both models.

Ecologically perhaps the most interesting results in Table 2 are the strong local pairwise correlations, which are not explained by null models. When interpreting the results, one should note that *p*-values of 1 are on rows where correlation count is zero in original data, meaning that they are not relevant. Fixed-fixed model considers correlations highly significant, while Rasch is more conservative, but also suggests that the correlation structure is significant and strong. For the NOW and NOW MN5 datasets this supports the previous findings: global structure seems to be explained by margin sums, while there still are significant local structures [1].

Results for dataset NOW MN5 also shed some light on swap chain patterns that were seen in the first experiment, which was also run with NOW MN5 dataset. In the first experiment we observed how swap chains converged differently depending on if they were started from the original dataset or the Rasch dataset. In Table 2, both fixed-fixed and Rasch models indicate that NOW MN5 has significantly high number of checkerboard units. They also show that the dataset has some significant and strong pairwise positive correlations on columns. For a pairwise positive correlation to exist, the two columns in question need to have low number of checkerboard units. The simultaneous existence of the two contradicting patterns implies that checkerboard units must be organized in a special way to make room for correlations. Swap operations shuffle the checkerboard units and quickly remove correlation structure, as can be seen from Fig 2 of first experiment. When Rasch model is used to generate a dataset similar to NOW MN5, it does not have the special organization of checkerboard units and swap operations do not have any major effect. The purpose of the null model is to preserve the high level structure of the dataset, as described by the margin sums, so the performance of the fixed-fixed models are undesirable in this case. The models generate very different null distributions, when they are given only slightly different margin sums as a starting point.

The general idea of using matrix sum based null models is to test statistical discoveries against trivial aspects of data. Some sites are more populated than others, as some of the species are more common than others. One is typically looking for patterns that are not explained by these simple features, but go beyond it. Highly optimistic figures in Table 2 would indicate that the fixed-fixed model failed to account for some of the margin sum driven features of data. Most importantly, in the experiment the choice of null model had dramatic effects on measures of significance. Finally, it is important to note that these experiments do not state that fixed-fixed models always, or even often, display undesired performance. In existing literature fixed-fixed models have been applied successfully in many instances, even with datasets that have relatively strong nested structure. The data analyst unfortunately cannot rely on their performance without considering the issues pointed out in this manuscript.

## Discussion

Using margin sums of a matrix to construct statistical significance tests is a difficult task. The manuscript discussed the established approach of using fixed-fixed margin sum constraints via the sequential swap procedure for significance testing and as an alternative presented stochastic constraints that constrain expected margin sums instead of strict sums. Major characteristics of both constraint families are summarized in Table 3.

**Table 3:** Summary of characteristics of fixed-fixed constraints and stochastic constraints (Rasch).

	Fixed-fixed constraints	Stochastic constraints
Maximum entropy model	sequential swaps	Rasch
Convergence	not known	trivial
Noise tolerance	tolerant	tolerant
Limitations	nested data	none known
Conservative	no	yes
Applications	empirical	empirical and analytical

Both families of constraints have corresponding maximum entropy models that implement the constraints without additional biases and allow efficient computational implementation. The sequential swap procedure still has the theoretically unsolved issue of convergence, but due to abundant availability of computing power, convergence is not of practical significance for sequential swaps. Both models have been found to be tolerant of noise (data not shown). Because the computational questions are largely solved, one might argue that the choice between the constraints seems to be purely ecological: it is up to the ecologist to choose the type of constraints that are better justified for the task at hand.

Ecological justifications for fixed-fixed margin sum constraints have been widely discussed in the literature and many of the original computational challenges have been resolved. The remaining challenge of judging the ecological implications of constraint family selection however requires both computational and ecological reasoning. Computational aspects still play a role because the ecological implications of fixed-fixed constraints cannot be evaluated without understanding the kind of patterns that are likely to emerge when the constraints are maintained. Fixed-fixed constraints have complex internal dependencies and those dependencies might dictate behavior that is undesired or surprising. The extreme examples are nested matrices, which cannot be permuted at all by the swap operations. Therefore the ecologist should ask if there are other cases where a certain matrix sum configuration leads to undesirable results.

The sequential swap procedure in itself is difficult to analyze closely enough to discover potential situations of undesirable behavior. However, experiments demonstrated cases where swaps converged to results that were counter-intuitive, likely due to “lock-in” effect, i.e., the permutation procedure being limited to a too strict set of matrices. The sequential swap method was sensitive to small changes in margin sums. With actual presence-absence datasets swap based tests gave highly optimistic indicators of significance.

The sequential swap procedure has been developed gradually in the context of presence-absence studies, though it was well known in computational sciences as well. One notable step of this development was the slightly incorrect original formulation of the algorithm and following discussion in literature, which first rejected the whole approach due to the detected bias (see [11] for a recount of the discussion and correct algorithm). One might describe the approach as bottom up: the model has been refined step by step as problems are discovered. The alternative approach is to work with a different set of constraints that allow analytical treatment and lead to a model with more predictable behavior. To that end, stochastic margin sum constraints were presented. Their computational implementation using the Rasch model is more suitable for analysis and has predictable computational implementation. As a consequence, Rasch allows one to derive independent cell specific probabilities and to directly calculate the probability of many patterns. For swaps it is not possible, because cell values are not independent due to fixed-fixed constraints.

The aspects of swap procedure demonstrated in this manuscript can be considered as a cautionary example about algorithmic null models: ingeniously simple methods can have complex dynamics and produce surprising results. Ecological reasoning should be the driving force for model development, but the computational manifestation should be such that it allows to cleanly express the assumptions without tangling them in to computational details. In practical terms, such work is often most successfully carried out in a cross-disciplinary collaboration.

Null models are tools for data analysts. The choice of tool always depends on the actual ecological application. To make an informed decision, the data analyst must be capable of understanding the implications of each alternative. Statistical tools should have understandable and predictable behavior. This manuscript demonstrated aspects of the sequential swap procedure that would surely come as a surprise to the data analyst.
